# Acute eGFR Changes and Their Mediation of Albuminuria Reduction with Empagliflozin and Finerenone

**DOI:** 10.1681/ASN.0000001071

**Published:** 2026-03-29

**Authors:** Rajiv Agarwal, Ricardo Correa-Rotter, Sankar D. Navaneethan, Kei Fukami, Hiddo J.L. Heerspink, Johannes F.E. Mann, Janet B. McGill, Amy K. Mottl, Masaomi Nangaku, Julio Rosenstock, Peter Rossing, Muthiah Vaduganathan, Charlie Scott, Li Li, Carolina Aldworth, Jennifer B. Green, Matthew R. Weir

**Affiliations:** 1 Division of Nephrology, Richard L. Roudebush VA Medical Center, Indiana University School of Medicine, Indianapolis, Indiana; 2 Department of Nephrology and Mineral Metabolism, National Medical Science and Nutrition Institute Salvador Zubirán, Mexico City, Mexico; 3 Baylor College of Medicine, Houston, Texas; 4 Division of Nephrology, Department of Medicine, Kurume University School of Medicine, Kurume, Japan; 5 Department of Clinical Pharmacy and Pharmacology, University Medical Center Groningen, University of Groningen, Groningen, The Netherlands; 6 KfH Kidney Centre, Munich, Germany; 7 Department of Nephrology and Hypertension, Friedrich Alexander University, Erlangen, Germany; 8 Division of Endocrinology, Metabolism and Lipid Research, School of Medicine, Washington University in St. Louis, St. Louis, Missouri; 9 University of North Carolina Kidney Center, UNC School of Medicine, Chapel Hill, North Carolina; 10 Division of Nephrology and Endocrinology, The University of Tokyo Graduate School of Medicine, Tokyo, Japan; 11 University of Texas Southwestern Medical Center, Dallas, Texas; 12 Steno Diabetes Center Copenhagen, Copenhagen, Denmark; 13 Department of Clinical Medicine, University of Copenhagen, Copenhagen, Denmark; 14 Division of Cardiovascular Medicine, Brigham and Women's Hospital, Harvard Medical School, Boston, Massachusetts; 15 Clinical Statistics and Analytics, Bayer US LLC, Whippany, New Jersey; 16 Global Medical Evidence, Bayer AG, Berlin, Germany; 17 US Medical Affairs, Bayer US LLC, Whippany, New Jersey; 18 Division of Endocrinology, Department of Medicine, Duke Clinical Research Institute, Duke University School of Medicine, Durham, North Carolina; 19 Division of Nephrology, Department of Medicine, University of Maryland School of Medicine, Baltimore, Maryland

**Keywords:** albuminuria, CKD, clinical trial, diabetes mellitus, diabetic kidney disease

## Abstract

**Key Points:**

- We investigated the effect of empagliflozin, finerenone, and their combination on eGFR decline in people with type 2 diabetes and albuminuria.
- Acute declines in eGFR occurred more in those on combination therapy, on diuretics, and with higher eGFR; eGFR changes were reversible.
- Finerenone's additive effect to empagliflozin on urinary albumin-to-creatinine ratio lowering was nonhemodynamic; empagliflozin lowered urinary albumin-to-creatinine ratio in part driven by eGFR change.

**Background:**

eGFR decline is common with sodium-glucose cotransporter 2 inhibitors and renin–angiotensin system inhibitors, often prompting treatment interruption or cessation, limiting cardiorenal benefits. This mostly prespecified COmbinatioN effect of FInerenone anD EmpaglifloziN in Participants with chronic kidney disease and type 2 diabetes using a UACR Endpoint (CONFIDENCE) trial analysis investigated the effect of empagliflozin, finerenone, and their combination on change from baseline in eGFR and its determinants and the relationship of change in eGFR with albuminuria reduction in people with type 2 diabetes and CKD.

**Methods:**

Evaluable participants (*N*=790) with type 2 diabetes, CKD, and albuminuria, receiving stable doses of renin–angiotensin system inhibitors, were randomized 1:1:1 to empagliflozin, finerenone, or both. The primary outcome was urinary albumin-to-creatinine ratio (UACR) change from baseline to day 180. We assessed mean eGFR change from baseline at day 14 (acute), determinants of acute eGFR decline, and acute eGFR change from baseline as a mediator of UACR reduction at day 180.

**Results:**

The mean acute eGFR decline was greater with combination therapy (−6.6 ml/min per 1.73 m^2^) than with finerenone (−2.1 ml/min per 1.73 m^2^) or empagliflozin (−4.8 ml/min per 1.73 m^2^) monotherapy; *P* < 0.001. Acute decline in eGFR was significantly more pronounced among participants with higher baseline eGFR and in those receiving diuretics at baseline (*P* < 0.001 for both factors). Baseline values for systolic BP and UACR had no statistically significant effect on acute eGFR decline. Exploratory analysis showed that acute eGFR change mediated 28% of the effect of adding empagliflozin to finerenone on UACR reduction at day 180 but only 5.2% of the effect when adding finerenone to empagliflozin. AKI was uncommon in all treatment groups.

**Conclusions:**

Acute eGFR decline was significantly associated with combination therapy, higher baseline eGFR, and diuretic use at baseline.

**Clinical Trial registry name and registration number:**

ClinicalTrials.gov, NCT05254002.

## Visual Abstract

**Figure:**
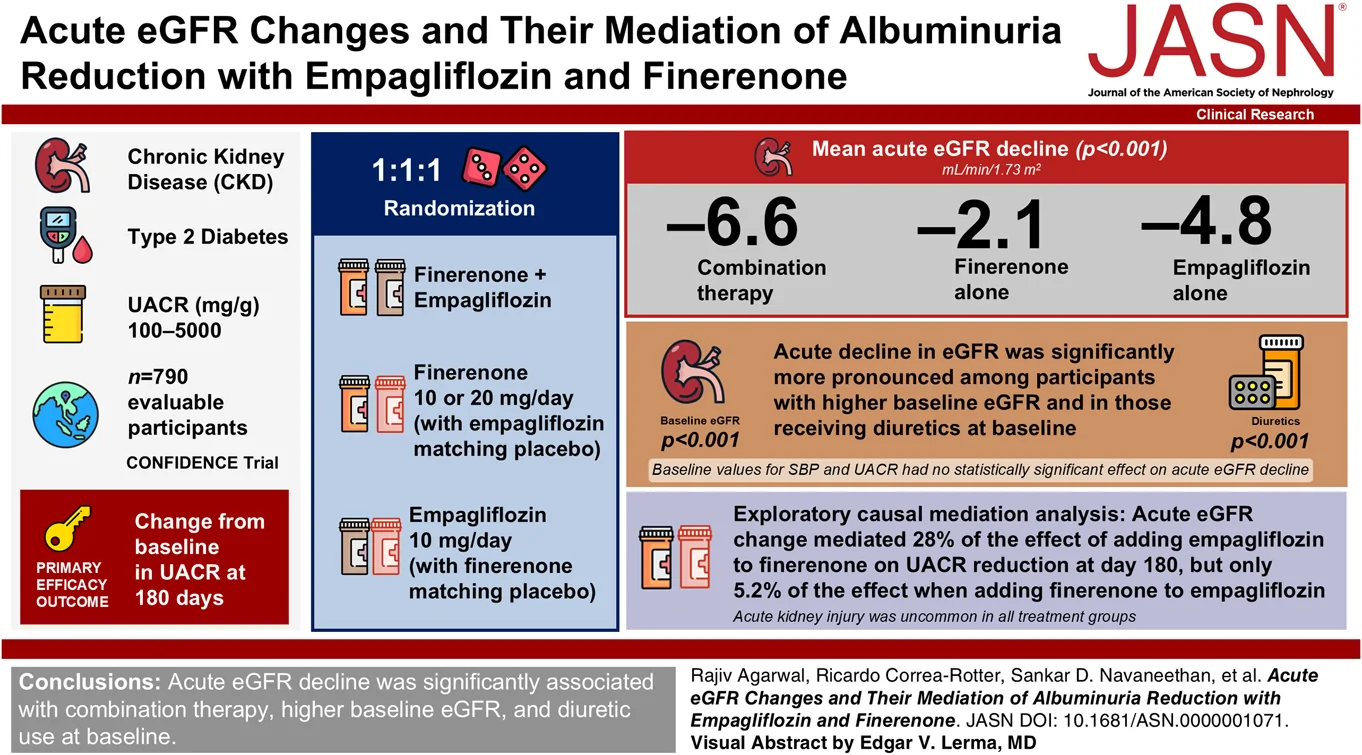

## Introduction

Since 2019, a growing number of therapeutic options have emerged for providing cardiorenal protection in people with type 2 diabetes and CKD. These include renin-angiotensin system inhibitors, sodium-glucose cotransporter 2 inhibitors (SGLT2is), finerenone (a nonsteroidal mineralocorticoid receptor antagonist), and glucagon-like peptide-1 receptor agonists.^1–6^ The COmbinatioN effect of FInerenone anD EmpaglifloziN in participants with chronic kidney disease and type 2 diabetes using a UACR Endpoint (CONFIDENCE) trial demonstrated that simultaneous therapy with the nonsteroidal mineralocorticoid receptor antagonist finerenone plus the SGLT2i empagliflozin led to a greater reduction in urinary albumin-to-creatinine ratio (UACR) than either treatment alone among participants with type 2 diabetes and CKD.^7^

The widespread adoption of these cardiorenal-beneficial therapies is hindered by a potential barrier: a decline in eGFR, particularly when multiple treatments are initiated simultaneously.^8^ This eGFR reduction can lead to the premature discontinuation of vital medications by both prescribers and patients.^9^

We conducted a prespecified analysis of the CONFIDENCE trial to investigate eGFR trajectories, the underlying determinants of eGFR change, and their potential association with clinical benefits, after initiating empagliflozin, finerenone, or both.^7,10,11^ Our analysis focused on two primary questions. First, we examined acute effects (from baseline to day 14), post-baseline eGFR trajectories, and determinants of eGFR change over time. Second, using exploratory causal mediation analysis, we explored whether the acute change in eGFR at day 14 mediated the reduction in UACR at day 180. This report details the results of these analyses, providing insights that may help guide treatment adherence and inform the long-term management of cardiorenal risk in people with type 2 diabetes.

## Methods

### Study Design and Participants

CONFIDENCE (NCT05254002) was a double-blind, phase 2, randomized, active-controlled trial conducted across 14 countries.^10,11^ The protocol, design, baseline characteristics, and primary results have been previously published.^7,10,11^

Eligible participants had type 2 diabetes with glycated hemoglobin levels of <11%, CKD with an eGFR between 30 and 90 ml/min per 1.73 m^2^, and persistent albuminuria, defined as a mean value of first morning void UACR between 100 and ≤5000 mg/g. All enrolled participants were required to be receiving a stable (>1 month), maximally tolerated dose of angiotensin-converting enzyme inhibitor or angiotensin-receptor blocker at baseline.^7,10^

Exclusion criteria included a diagnosis of type 1 diabetes, a screening serum potassium level of >4.8 mmol/L, symptomatic heart failure with reduced ejection fraction, or those who had had a stroke or myocardial infarction or had been hospitalized for worsening heart failure within 90 days before the screening visit. Patients who received an SGLT2i or a potassium-binding agent 8 weeks before screening were also excluded.^7,10^

The trial was approved by the institutional review board at each study site and conducted in accordance with the principles of the Declaration of Helsinki. All participants provided written informed consent.

### Interventions

Participants were randomized in a 1:1:1 ratio to receive empagliflozin alone, finerenone alone, or the combination of both. Empagliflozin was dosed at 10 mg per day. Finerenone was initiated at 20 mg per day when the baseline eGFR was ≥60 ml/min per 1.73 m^2^ and at 10 mg per day when the baseline eGFR was <60 ml/min per 1.73 m^2^. Randomization was stratified according to eGFR (<60 and ≥60 ml/min per 1.73 m^2^) and UACR (≤850 and >850 mg/g) measurements at the screening visit. The trial group assignments were concealed from the investigators, the treating physicians, the participants, and the outcome assessors using a double-dummy trial design.

### Outcome Measures and Definitions

The primary efficacy outcome was the relative change in the log-transformed mean UACR from baseline to day 180. Secondary outcomes included change from baseline in eGFR.^7^

In this analysis, we report the eGFR decline as a decrease from baseline of ≥30% at any visit during study treatment. In addition, investigator-reported AKI, spontaneously reported as adverse events irrespective of the eGFR cutoff, were prospectively collected. The safety analysis set, comprising all randomized participants who received at least one dose of study medication, was used. For each longitudinal and mediation analysis reported here, all individuals in the safety analysis set with available data at the relevant time points were included.

### Statistical Analysis

For the purposes of this analysis, acute change in eGFR was defined as the change from baseline to day 14, corresponding to the earliest available postbaseline measurement. Subsequent changes after day 14 were classified as the eGFR trajectory. Patients who received at least one dose of study drug were included in this analysis.

#### Linear Mixed Models for eGFR Change

A linear mixed model^12^ was used to estimate determinants of the mean acute change in eGFR (from baseline to day 14) and the trajectory of change (at any time after baseline). The first post-baseline measurement of eGFR (day 14) was set as the reference level. Fixed effects included treatment (finerenone, empagliflozin, or combination therapy), visit (coded as a categorical variable), treatment×visit interaction, baseline eGFR, baseline eGFR×visit, log of baseline UACR, log baseline UACR×visit, baseline systolic BP, baseline systolic BP×visit interaction, baseline diuretic use, and baseline diuretic use×visit interaction. Estimates that included the covariate×visit interaction provided information on the eGFR trajectory; estimates without this interaction provided the determinants of acute change in eGFR. These fixed effects were selected based on biologic plausibility that these variables could influence eGFR and its trajectory. Random effects included participant and visits with an unstructured covariance matrix. Maximal likelihood estimates were used to derive the estimated means and SEMs. Wald tests were used to assess the significance of any effects on acute change in eGFR.

To facilitate interpretation and visualization of the data, the results are presented as marginal means from the linear mixed models with the covariates in the model, modeled at their mean values.

#### Logistic Regression Models for eGFR Change

Logistic regression models were used to assess the odds of having ≥30% eGFR decline from baseline across treatment groups. The outcome variable was ≥30% eGFR decline at any visit during the study treatment period (up to day 180). Covariates included treatment and baseline log-transformed UACR, eGFR, diuretic use, and systolic BP.

#### Effect of Acute eGFR Change on Subsequent Outcome of UACR Reduction

Exploratory causal mediation analysis evaluated whether acute eGFR change mediated treatment effects on change from baseline in log UACR at day 180.^13^ We followed the methodologic assumptions underlying causal mediation analysis proposed by Lee *et al*.^13^ To enable inclusion, participants required paired data points for both eGFR (baseline and day 14) and UACR (baseline and day 180). Linear regression quantified treatment effects on the mediator (observed acute change in eGFR) and the outcome (change in log UACR from baseline to day 180). Randomization stratification variables (baseline eGFR and UACR strata) were included in both models. Natural direct (independent of the mediator), natural indirect (dependent on the mediator), and total effects, as well as percent mediation, were estimated, and 1000 bootstrap replicates were used to determine the associated 95% confidence intervals (CIs). A directed acyclic graph representing the mediation model is shown as Supplemental Figure 1.^13^

#### Adverse Event Analysis

Treatment-emergent adverse events, defined as adverse events occurring within 3 days of the last study drug dose, were summarized descriptively by subgroup. Specific adverse events, including hyperkalemia, hypotension, and AKI, were also summarized descriptively.

All statistical analyses were performed using Stata 19.5 (StataCorp, College Station, TX).

## Results

A total of 790 participants were included in this analysis, of whom 265 received combination therapy, 263 received finerenone, and 262 received empagliflozin. Baseline eGFR data were available for all except four of these individuals.

### Mean Changes in eGFR

**Figure 1:**
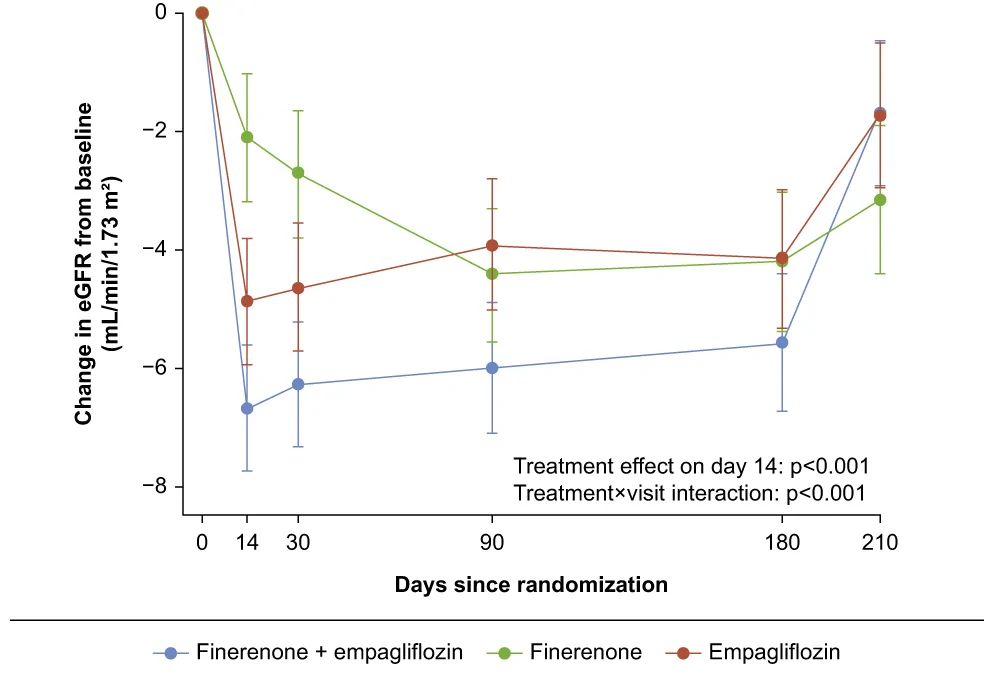
**Mean changes in eGFR from baseline by the treatment group.** The figure displays the mean change in eGFR from baseline to day 210 by the treatment group. The marginal mean changes are calculated for covariates at their means. Error bars represent 95% CI. Treatment was stopped at day 180. CI, confidence interval.

The mean changes in eGFR from baseline (Supplemental Table 1) are shown graphically in Figure 1. There was a statistically significant effect of treatment on the mean change in eGFR from baseline (*P* < 0.001). The acute change in eGFR was −6.6 ml/min per 1.73 m^2^ (95% CI, −7.7 to −5.6) in the combination group, −2.1 ml/min per 1.73 m^2^ (95% CI, −3.2 to −1.1) in the finerenone group, and−4.8 ml/min per 1.73 m^2^ (95% CI, −5.9 to −3.8) in the empagliflozin group. The immediacy of eGFR decline in the combination and empagliflozin groups resulted in nadir values at day 14. In comparison, the trajectory of eGFR decline in the finerenone group was much less steep, with the nadir not reached until day 90 (−4.4 ml/min per 1.73 m^2^ [95% CI, −5.5 to −3.3]).

### Determinants of the Acute Mean Changes in eGFR and the Trajectory

**Figure 2:**
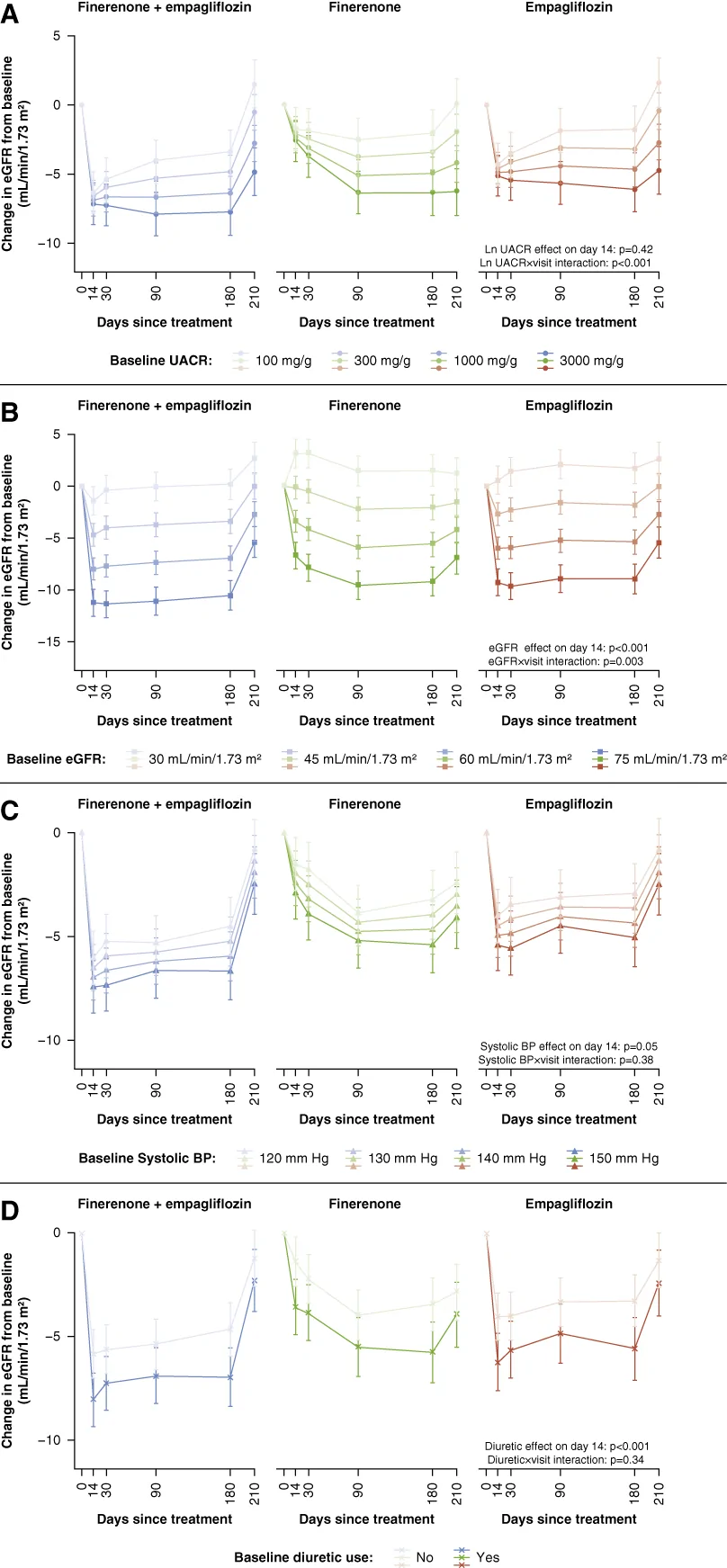
**Mean changes in eGFR from baseline.** (A) Baseline UACR, (B) baseline eGFR, (C) baseline systolic BP, and (D) baseline diuretic use. The figure displays the mean change in eGFR from baseline to day 210 over increasing levels of (A) baseline UACR, (B) baseline eGFR, (C) baseline systolic BP, and (D) baseline diuretic use, indicated by progressively deeper colors over each treatment group. The marginal mean changes are calculated for covariates at their means. Error bars represent 95% CI. Treatment was stopped at day 180. The change from baseline in eGFR at day 14 and the trajectory of eGFR were statistically significantly modified by baseline eGFR regardless of treatment. The change from baseline in eGFR at day 14 was modified by baseline systolic BP regardless of treatment at a borderline level of statistical significance (*P* = 0.053). The trajectory of eGFR was not modified by the baseline level of systolic BP (*P* = 0.38). The change from baseline in eGFR at day 14 was statistically significantly modified by baseline diuretic use regardless of treatment (*P* = 0.0006). However, the diuretic×visit effect was not statistically significant, indicating that the baseline diuretic use did not modify the trajectory of eGFR. Ln, natural log; UACR, urinary albumin-to-creatinine ratio.

Besides treatment, the following two factors significantly influenced the acute change in eGFR: baseline levels of eGFR and baseline diuretic use. A higher baseline UACR was not a statistically significant determinant of the acute eGFR decline (*P* = 0.42) but was a determinant of the subsequent chronic trajectory (*P* < 0.001; Figure 2A and Supplemental Table 2). A higher baseline eGFR was a statistically significant determinant of both the acute eGFR decline (*P* < 0.001) and the subsequent chronic trajectory (*P* = 0.003; Figure 2B and Supplemental Table 3). Participants with higher baseline eGFR experienced more pronounced declines in eGFR, consistent with the “regression to the mean” phenomenon. Furthermore, among those with eGFR of 30 ml/min per 1.73 m^2^, we did not observe a large and persistent decline in eGFR in any treatment group. Participants with a higher baseline level of systolic BP had a marginally greater acute reduction in eGFR (*P* = 0.05) but systolic BP was not associated with the eGFR trajectory (*P* = 0.38; Figure 2C and Supplemental Table 4). Finally, compared with participants not receiving diuretics, those prescribed diuretics at baseline experienced a statistically significantly greater acute decline in eGFR (*P* < 0.001; Figure 2D and Supplemental Table 5). However, baseline diuretic use did not statistically significantly modify the long-term eGFR trajectory after the acute change (*P* = 0.34). The mean reduction in eGFR was reversible and returned toward baseline after drug withdrawal.

### Baseline Characteristics and the Odds of eGFR Decline

Participant baseline characteristics by the occurrence of an eGFR decline of ≥30% at any visit during the treatment period are presented in Table 1.

**Table 1:** Participant baseline characteristics by eGFR decline of ≥30%

Clinical Characteristic	eGFR Decline from Baseline ≥30% at Any Visit during Study Treatment		Total
	Yes	No	
Sample size, *n* (%)	133 (17)	657 (83)	790 (100)
**Treatment group, *n* (%)**			
Combination therapy	59 (44)	206 (31)	265 (34)
Finerenone	45 (34)	218 (33)	263 (33)
Empagliflozin	29 (22)	233 (35)	262 (33)
Age, yr, mean (SD)	66 (10)	67 (10)	67 (10)
Male sex, *n* (%)	97 (73)	495 (75)	592 (75)
**Geographic region, *n* (%)**			
Asia	57 (43)	298 (45)	355 (45)
Europe	33 (25)	180 (27)	213 (27)
North America	43 (32)	179 (27)	222 (28)
BMI, kg/m^2^, mean (SD)	29.4 (6.3)	29.3 (6.0)	29.3 (6.0)
Serum potassium, mmol/L, mean (SD)	4.4 (0.4)	4.5 (0.4)	4.5 (0.4)
Systolic BP, mm Hg, mean (SD)	137 (13)	135 (13)	135 (13)
History of atherosclerotic cardiovascular disease, *n* (%)	41 (32)	179 (28)	220 (28)
eGFR, ml/min per 1.73 m^2^, mean (SD)	59 (18)	53 (17)	54 (17)
**KDIGO CKD stage, *n* (%)**			
G1 or G2	63 (47)	209 (32)	272 (35)
G3A	37 (28)	191 (29)	228 (29)
G3B	32 (24)	227 (35)	259 (33)
G4	1 (0.8)	26 (4.0)	27 (3.4)
UACR, mg/g, median (25%–75%)	663 (298–1185)	557 (292–1070)	583 (292–1096)
**Severity of albuminuria, *n* (%)**			
<300 mg/g	33 (25)	169 (26)	202 (26)
300 to <1000 mg/g	59 (44)	312 (47)	371 (47)
≥1000 mg/g	41 (31)	176 (27)	217 (27)
HbA1c, %, mean (SD)	7.3 (1.3)	7.3 (1.2)	7.3 (1.2)
**Concomitant medications, *n* (%)**			
ACEi or ARB	131 (98)	646 (98)	777 (98)
Statins	102 (77)	489 (74)	591 (75)
Diuretics	67 (50)	219 (33)	286 (36)
Insulin	50 (38)	265 (40)	315 (40)
GLP-1 RA	25 (19)	153 (23)	178 (23)

ACEi, angiotensin-converting enzyme inhibitor; ARB, angiotensin receptor blocker; BMI, body mass index; GLP-1 RA, glucagon-like peptide-1 receptor agonist; HbA1c, glycated hemoglobin; KDIGO, Kidney Disease Improving Global Outcomes; UACR, urinary albumin-to-creatinine ratio.

Overall, an eGFR decline of ≥30% at any visit during treatment occurred in 133/790 participants (17%): 59/265 (22%) in the combination therapy group, 45/263 (17%) in the finerenone group, and 29/262 (11%) in the empagliflozin group. In line with the findings described above, participants who developed eGFR declines of ≥30% had a higher baseline eGFR and a greater diuretic use at baseline compared with individuals who did not experience such an eGFR decline.

An analysis of the number of participants with a ≥30% decline from baseline in eGFR at any visit during treatment showed increasing numbers of events over time in each of the three treatment groups, but more so in the combination therapy group (Table 2 and Supplemental Figure 2).

**Table 2:** Odds of eGFR decline ≥30% at any visit during study treatment

Clinical Variable	OR (95% CI)
**Treatment group**	
Combination therapy group	Reference
Combination therapy versus finerenone	1.42 (0.90 to 2.22)
Combination therapy versus empagliflozin	2.35 (1.42 to 3.90)
Baseline UACR (per log higher mg/g)	1.26 (1.02 to 1.54)
Baseline eGFR (per 10 ml/min per 1.73 m^2^ higher)	1.24 (1.11 to 1.39)
**Diuretic use (none)**	Reference
Diuretic use at baseline	1.96 (1.32 to 2.91)
Baseline systolic BP (per 10 mm Hg higher)	1.06 (0.92 to 1.24)

CI, confidence interval; OR, odds ratio; UACR, urinary albumin-to-creatinine ratio.

Combination therapy was associated with greater odds of an eGFR decline ≥30% at any visit during treatment compared with empagliflozin alone (odds ratio [OR], 2.35; 95% CI, 1.42 to 3.90). Similarly, combination therapy was associated with an OR of 1.42 (95% CI, 0.90 to 2.22) compared with finerenone alone. Other independent determinants of an eGFR decline ≥30% at any visit during treatment were baseline UACR (OR per log change mg/g, 1.26; 95% CI, 1.02 to 1.54), baseline eGFR (OR per 10 ml/min per 1.73 m^2^ change, 1.24; 95% CI, 1.11 to 1.39), and baseline diuretic use (OR, 1.96; 95% CI, 1.32 to 2.91). Baseline systolic BP was not an independent determinant of an eGFR decline ≥30%.

Despite the higher frequency of eGFR decline ≥30% at any visit during treatment in the combination therapy arm, investigator-reported AKI events were rare across all treatment groups (see Adverse Event Analysis by eGFR Decline ≥30% section below).

### Causal Mediation Analysis of Acute eGFR Change and UACR Reduction

A total of 690 participants had the required eGFR and UACR data for inclusion in the exploratory mediation analysis, and the results are presented in Table 3.

**Table 3:** Mediation analysis of acute eGFR change on urinary albumin-to-creatinine ratio reduction at day 180

Effect	Percent Reduction UACR (95% CI)	
	Combination Therapy versus Finerenone	Combination Therapy versus Empagliflozin
Total effect	−29 (−40 to −15)	−32 (−42 to −20)
Independent of eGFR (NDE)	−21 (−35 to −5.1)	−30.3 (−41 to −18)
Mediated by eGFR (NIE)	−9.0 (−17 to −0.5)	−2.0 (−5.0 to +1.2)
Percent mediated	28 (−1.8 to 58), *P* = 0.07	5.2 (−3.3 to 14), *P* = 0.23

CI, confidence interval; NDE, natural direct effect; NIE, natural indirect effect; UACR, urinary albumin-to-creatinine ratio.

At day 180, the reduction in UACR with combination therapy was 29% (95% CI, 15 to 40) greater than with finerenone alone. Of this UACR reduction, approximately 21% (95% CI, 5.1 to 35) was independent of the acute eGFR change, while 9.0% (95% CI, 0.5 to 17) was dependent on it. Overall, the acute change in eGFR mediated 28% of the effect of adding empagliflozin to finerenone (*P* = 0.07).

At day 180, the reduction in UACR was 32% (95% CI, 20 to 42) greater with combination therapy than with empagliflozin alone. Of this reduction, approximately 30% (95% CI, 18 to 41) was independent of the acute change in eGFR, while 2.0% (95% CI, −1.2 to 5.0) was dependent on it. Overall, the acute change in eGFR mediated 5.2% of the effect of adding finerenone to empagliflozin (*P* = 0.23).

### Adverse Event Analysis by eGFR Decline ≥30%

Table 4 presents the adverse events by eGFR decline ≥30% at any visit during treatment, stratified by treatment group. Symptomatic hypotension occurred in three participants (0.4%), all in the combination therapy group. AKI occurred in four participants (0.5%): two in the combination therapy group and two in the finerenone group. Genital mycotic infections were seen in eight participants (1.0%): four in the combination therapy group and four in the empagliflozin group. Hyperkalemia was observed in 113/790 (14%) participants and was almost twice as common when eGFR declined by ≥30% at any visit during treatment (31/133 participants, 23%) compared with when it did not (82/657 participants, 12%). This pattern was independent of treatment received. The higher incidence of hyperkalemia among participants with versus without eGFR decline ≥30% was particularly marked when considering severe cases (serum potassium >6.0 mmol/L), with frequencies of 11% and a >4-fold higher frequency. The higher incidence among individuals with versus without eGFR decline ≥30% was less pronounced (approximately 1.5-fold) with respect to moderate cases (serum potassium >5.5 mmol/L).

**Table 4:** Safety events by eGFR decline ≥30%

Event, *n* (%)	eGFR Decline from Baseline ≥30% at Any Visit during Study Treatment		Total
	Yes	No	
**Symptomatic hypotension**	1/133 (0.8)	2/657 (0.3)	3/790 (0.4)
Combination therapy	1/59 (1.7)	2/206 (1.0)	3/265 (1.1)
Finerenone	0/45 (0.0)	0/218 (0.0)	0/263 (0.0)
Empagliflozin	0/29 (0.0)	0/233 (0.0)	0/262 (0.0)
**AKI**	2/133 (1.5)	2/657 (0.3)	4/790 (0.5)
Combination therapy	1/59 (1.7)	1/206 (0.5)	2/265 (0.8)
Finerenone	1/45 (2.2)	1/218 (0.5)	2/263 (0.8)
Empagliflozin	0/29 (0.0)	0/233 (0.0)	0/262 (0.0)
**Genital mycotic infection**	1/133 (0.8)	7/657 (1.1)	8/790 (1.0)
Combination therapy	1/59 (1.7)	3/206 (1.5)	4/265 (1.5)
Finerenone	0/45 (0.0)	0/218 (0.0)	0/263 (0.0)
Empagliflozin	0/29 (0.0)	4/233 (1.7)	4/262 (1.5)
**Urosepsis**	1/133 (0.8)	1/657 (0.2)	2/790 (0.3)
Combination therapy	1/59 (1.7)	0/206 (0.0)	1/265 (0.4)
Finerenone	0/45 (0.0)	0/218 (0.0)	0/263 (0.0)
Empagliflozin	0/29 (0.0)	1/233 (0.4)	1/262 (0.4)
**Hyperkalemia**	31/133 (23)	82/657 (12)	113/790 (14)
Combination therapy	15/59 (25)	25/206 (12)	40/265 (15)
Finerenone	11/45 (24)	37/218 (17)	48/263 (18)
Empagliflozin	5/29 (17)	20/233 (8.6)	25/262 (9.5)
**Moderate hyperkalemia^a^**	24/133 (18)	74/657 (11)	98/790 (12)
Combination therapy	11/59 (19)	23/206 (11)	34/265 (12)
Finerenone	8/45 (18)	35/218 (16)	43/263 (16)
Empagliflozin	5/29 (17)	16/233 (76.9)	21/262 (8.0)
**Severe hyperkalemia^b^**	15/133 (11)	16/657 (2.4)	31/790 (3.9)
Combination therapy	8/59 (14)	4/206 (1.9)	12/265 (4.5)
Finerenone	6/45 (13)	6/218 (2.8)	12/263 (4.6)
Empagliflozin	1/29 (3.4)	6/233 (2.6)	7/262 (2.7)

a Serum potassium >5.5 mmol/L.

b Serum potassium >6.0 mmol/L.

## Discussion

These analyses of the CONFIDENCE trial, which were prespecified except for the mediation analysis, provide critical insights into the participant-level factors that modify the eGFR trajectory in response to finerenone, empagliflozin, and combination therapy. Our findings clearly distinguish between determinants of the acute, hemodynamic eGFR decline and those influencing the change over 6 months. The magnitude of the acute eGFR decline appears to be driven by factors related to hemodynamic reserve and volume status: *i.e*., the acute eGFR decline was greater in participants with higher baseline eGFR and those receiving diuretics at baseline. A prior analysis of 53 trials comprising 56,413 participants noted a greater eGFR decline when baseline eGFR was higher.^14^ Notably, in our analysis, this acute eGFR decline was absent among participants with a baseline eGFR of 30 ml/min per 1.73 m^2^, suggesting a blunted hemodynamic response in those with advanced CKD. Conversely, the eGFR trajectory over 6 months was modified by baseline levels of UACR and eGFR, which are classic indicators of underlying disease severity and progression risk.^8^ The reversible eGFR decline and subsequent return toward baseline following withdrawal of study medication provides reassurance that the acute reductions do not represent permanent nephron loss. This distinction reinforces the idea that while the acute eGFR decline is a manageable hemodynamic effect, the subsequent therapeutic benefit on the eGFR slope is linked to the patient's underlying risk profile.^8^

Compared with empagliflozin alone, combination therapy more than doubled the odds of an eGFR decline ≥30%. The acute eGFR dip in the combination arm represents an expected, functional, hemodynamic signature primarily driven by the SGLT2i. The incidence of AKI events was low, consistent with previous observations that SGLT2i-associated eGFR declines are driven by tubuloglomerular feedback adaptation, and that finerenone use is not associated with AKI.^15,16^ Thus, the occurrence of acute eGFR dip in recipients of combination therapy does not imply structural harm from the addition of finerenone. The reversible nature of the decline in eGFR supports the notion of functional “unloading” of the glomerulus, similar to the effects observed with renin–angiotensin system inhibitors.

In cases where a significant eGFR decline occurs, clinicians should first consider extrinsic factors such as nonsteroidal anti-inflammatory drug use, volume depletion, or excessive diuretic dosing before discontinuing disease-modifying therapies.^8,17,18^ Prescribers should proactively inform patients that a slight increase in serum creatinine (or a decline in eGFR) is an expected sign that the medication is “unloading” the pressure in the kidney.^8,18,19^ The pattern of adverse events suggests that hyperkalemia and eGFR declines coincide. Compared with participants who did not have an eGFR decline ≥30%, those with such a decline showed an almost two-fold higher incidence of hyperkalemia. The higher incidence of hyperkalemia among individuals with eGFR decline ≥30% was more marked (>4-fold) when considering severe cases (serum potassium >6.0 mmol/L). The hyperkalemia incidence rates suggest that, among recipients without eGFR decline ≥30%, the addition of empagliflozin to finerenone may provide a degree of protection against the risk of hyperkalemia associated with nonsteroidal mineralocorticoid receptor antagonist therapy. In practice, careful monitoring of potassium would be prudent in patients with an eGFR decline ≥30% following the initiation of treatment with finerenone, either alone or with an SGLT2i. We would suggest monitoring throughout the first 90 days after treatment initiation to assess early effects on both eGFR and serum potassium.

Our exploratory causal mediation analysis showed that the mechanisms for UACR reduction differed markedly between empagliflozin and finerenone. When comparing combination therapy with finerenone alone, the 29% greater UACR reduction was partially mediated by the acute change in eGFR (percent mediated: 28%). This suggests that a notable portion of the additive benefit from adding empagliflozin is linked to its acute hemodynamic effect. By contrast, when compared with empagliflozin alone, the 32% greater UACR reduction with combination therapy was almost entirely independent of the acute eGFR change (percent mediated: 5.2%). This finding suggests that the additive benefit of finerenone is not hemodynamically mediated but possibly stems from other mechanisms, such as its direct anti-inflammatory and antifibrotic effects. Owing to the exploratory nature of the mediation analysis, wide CIs, and *P* values above 0.05, the results should be interpreted with a degree of caution. However, they are supported by preclinical data showing that SGLT2 deficiency prevents glomerular hyperfiltration but does not attenuate markers of kidney fibrosis or inflammation.^20^

There are some limitations of the current analysis. As an exploratory analysis of a randomized trial, the clinical determinants of eGFR change, either acutely or over 6 months, while physiologically plausible, should not be construed as causal in nature. Residual confounding and chance findings remain possible, particularly considering the *post hoc* study design and the wide CIs associated with the mediation analysis. Furthermore, the study was limited in its duration, and it was not designed to evaluate the long-term decline in eGFR.

In conclusion, the acute eGFR decline observed with combination therapy of empagliflozin and finerenone may be interpreted as an expected pharmacodynamic consequence of glomerular hemodynamic unloading, rather than an indicator of kidney injury. This is supported by the exploratory causal mediation analysis, which links the eGFR decline to positive long-term UACR reduction. Crucially, the absence of this eGFR decline in patients with advanced CKD suggests acceptable safety in more advanced disease stages. Nevertheless, an eGFR decline ≥30% should alert clinicians to the need for more vigilant monitoring of serum potassium, given that our analysis suggests the risk of hyperkalemia among patients experiencing an eGFR decline may be nearly doubled. Combined use of a nonsteroidal mineralocorticoid receptor antagonist and an SGLT2i may be beneficial in addressing the multifaceted nature of CKD progression in type 2 diabetes.

## Supplementary Material

## Data Availability

Original data generated for the study will be made available upon reasonable request to the corresponding author. Data Type: Clinical Trial Data. Reason for Restricted Access: Availability of the data underlying this publication will be determined according to Bayer’s commitment to the European Federation of Pharmaceutical Industries and Associations/Pharmaceutical Research & Manufacturers Association “Principles for responsible clinical trial data sharing.” This pertains to scope, timepoint, and process of data access. As such, Bayer commits to sharing upon request from qualified scientific and medical researchers, patient-level clinical trial data, study-level clinical trial data, and protocols from clinical trials in patients for medicines and indications approved in the United States and European Union as necessary for conducting legitimate research. This applies to data on new medicines and indications that have been approved by the European Union and United States regulatory agencies on or after January 1, 2014. Interested researchers can use www.vivli.org to request access to anonymized patient-level data and supporting documents from clinical studies to conduct further research that can help advance medical science or improve patient care. Information on the Bayer criteria for listing studies and other relevant information is provided in the member section of the portal. Data access will be granted to anonymized patient-level data, protocols, and clinical study reports after approval by an independent scientific review panel. Bayer is not involved in the decisions made by the independent review panel. Bayer will take all necessary measures to ensure that patient privacy is safeguarded.

## Declarative Statements

This study includes clinical experimentation and received Institutional Review Board or Ethics Committee approval. All patients provided written informed consent. This study includes clinical experimentation and complies with the Declaration of Helsinki.

## Supplemental Material

Supplemental Figure 1. eGFR decline as a mediator of UACR lowering.

Supplemental Figure 2. Participants with eGFR decline ≥30% from baseline.

Supplemental Table 1. Change in eGFR from baseline within each treatment group.

Supplemental Table 2. Change in eGFR from baseline within each treatment group by UACR.

Supplemental Table 3. Change in eGFR from baseline within each treatment group by eGFR.

Supplemental Table 4. Change in eGFR from baseline within each treatment group by systolic BP.

Supplemental Table 5. Change in eGFR from baseline within each treatment group by diuretic use at baseline.
