# Supplementary material for: Acute eGFR Changes and Their Mediation of Albuminuria Reduction with Empagliflozin and Finerenone
Source: J Am Soc Nephrol. 2026 Mar 29;37(9):1969–78. doi: 10.1681/ASN.0000001071 (PMC13567893; doi:10.1681/ASN.0000001071)
Supplement: Supplementary file 1 [file jasn-37-1969-s001.pdf]

ASN American Society of Nephrology
JASN Journal of the American Society of Nephrology
KIDNEY360 Accessing Our World From Every Angle
CJASN Clinical Journal of the American Society of Nephrology

# ASN Journal Disclosure Form

As per ASN journal policy, I have disclosed any financial relationships or commitments I have held in the past 36 months as included below. I have listed my Current Employer below to indicate there is a relationship requiring disclosure. If no relationship exists, my Current Employer is not listed.

R. Agarwal reports the following:
Employer: Veteran's Administration (part time); Consultancy: Akebia, Bayer, Boehringer Ingelheim, Eli Lilly, Chinook, Alnylam, Vertex, Intercept Pharmaceuticals, Novartis; Honoraria: Akebia, Bayer, Boehringer Ingelheim, Chinook, Vertex, Intercept Pharmaceuticals, Novartis, Alnylam; Patents or Royalties: UpToDate; and Advisory or Leadership Role: Hypertension, NDT, Akebia, Bayer, Boehringer Ingelheim, Chinook, Vertex, Novartis, Alnylam.

I understand that the information above will be published within the journal article, if accepted, and that failure to comply and/or to accurately and completely report the potential financial conflicts of interest could lead to the following: 1) Prior to publication, article rejection, or 2) Post-publication, sanctions ranging from, but not limited to, issuing a correction, reporting the inaccurate information to the authors' institution, banning authors from submitting work to ASN journals for varying lengths of time, and/or retraction of the published work.

Name: Rajiv Agarwal
Manuscript ID: JASN-2026-000059R2
Manuscript Title: Acute eGFR Changes and Their Mediation of Albuminuria Reduction with Empagliflozin and Finerenone
Date of Completion: February 26, 2026
Disclosure Updated Date: July 25, 2025

ASN American Society of Nephrology
JASN Journal of the American Society of Nephrology
KIDNEY360 Accessing Our World From Every Angle
CJASN Clinical Journal of the American Society of Nephrology

# ASN Journal Disclosure Form

As per ASN journal policy, I have disclosed any financial relationships or commitments I have held in the past 36 months as included below. I have listed my Current Employer below to indicate there is a relationship requiring disclosure. If no relationship exists, my Current Employer is not listed.

C. Aldworth reports the following:
Employer: Bayer Corporation; and Ownership Interest: Bayer.

I understand that the information above will be published within the journal article, if accepted, and that failure to comply and/or to accurately and completely report the potential financial conflicts of interest could lead to the following: 1) Prior to publication, article rejection, or 2) Post-publication, sanctions ranging from, but not limited to, issuing a correction, reporting the inaccurate information to the authors' institution, banning authors from submitting work to ASN journals for varying lengths of time, and/or retraction of the published work.

Name: Carolina A R Aldworth
Manuscript ID: JASN-2026-000059
Manuscript Title: Acute eGFR Changes and Their Mediation of Albuminuria Reduction with Empagliflozin and Finerenone: A CONFIDENCE Trial Analysis
Date of Completion: January 15, 2026
Disclosure Updated Date: January 15, 2026

ASN American Society of Nephrology
JASN Journal of the American Society of Nephrology
KIDNEY360 Accessing Our World From Every Angle
CJASN Clinical Journal of the American Society of Nephrology

# ASN Journal Disclosure Form

As per ASN journal policy, I have disclosed any financial relationships or commitments I have held in the past 36 months as included below. I have listed my Current Employer below to indicate there is a relationship requiring disclosure. If no relationship exists, my Current Employer is not listed.

R. Correa-Rotter reports the following:
Employer: Instituto Nacional de Ciencias Médicas y Nutrición Salvador Zubiran, MEXICO  and; Universidad Nacional Autónoma de México, MEXICO; Consultancy: Astra Zeneca, GSK, Boheringer Ingelheim, Bayer, Chinook, Novonordisk; Research Funding: Astra Zeneca, Novonordisk, Roche, Chinook, Lilly; Honoraria: Amgen, Astra Zeneca,  Boehringer Ingelheim, Sanofi, Bayer, Amgen, Lilly; Advisory or Leadership Role: National Leader ASCEND study, GSK; National Leader  and International Expert Forum FLOW study, Novonordisk, membership steering Committee FINE-REAL, Bayer. Editorial Board Nefrologia Latinoamericana, American Journal of Kidney Diseases, and Associate Editor: Blood Purification. Member of the Steering Committee of the World Kidney Day organization.; Speakers Bureau: Amgen, Astra Zeneca,  Boehringer Ingelheim, Abbvie, Sanofi, Bayer, Novonordisk, Lilly; and Other Interests or Relationships: Member of ASN, of International Society of Nephrology; Member of National Kidney Foundation; Member Mexican Institute for Research in Nephrology; Member Latin American Society of Nephrology and Hypertension; Member ERA.

I understand that the information above will be published within the journal article, if accepted, and that failure to comply and/or to accurately and completely report the potential financial conflicts of interest could lead to the following: 1) Prior to publication, article rejection, or 2) Post-publication, sanctions ranging from, but not limited to, issuing a correction, reporting the inaccurate information to the authors' institution, banning authors from submitting work to ASN journals for varying lengths of time, and/or retraction of the published work.

Name: Ricardo Correa-Rotter
Manuscript ID: JASN-2026-000059R2
Manuscript Title: Acute eGFR Changes and Their Mediation of Albuminuria Reduction with Empagliflozin and Finerenone
Date of Completion: February 27, 2026
Disclosure Updated Date: November 21, 2025

ASN American Society of Nephrology | JASN Journal of the American Society of Nephrology | KIDNEY360 Accessing Our World From Every Angle | CJASN Clinical Journal of the American Society of Nephrology

## ASN Journal Disclosure Form

As per ASN journal policy, I have disclosed any financial relationships or commitments I have held in the past 36 months as included below. I have listed my Current Employer below to indicate there is a relationship requiring disclosure. If no relationship exists, my Current Employer is not listed.

K. Fukami reports the following:
Employer: Kurume University School of Medicine; Consultancy: Boehringer ingelheim, Ono pharmaceutical, Astrazeneca  pharmaceutical, Bayer pharmaceutical, Takeda Pharmaceuticals, Sanofi, Sumitomo Pharma, ZERIA Pharmaceutical;; Honoraria: Kyowa-Kirin pharmaceutical, Boehringer ingelheim, Ono pharmaceutical, Astrazeneca  pharmaceutical, Bayer pharmaceutical, GSK Co. Inc, Mitsubisi Pharma Co. Otsuka Pharmaceutical Co., Ltd., Eli Lilly and Company, Takeda Pharmaceuticals, Sanofi, Sumitomo Pharma, Kissei Pharmaceutical Co., Ltd, Nobelpharma Co., Ltd., ZERIA Pharmaceutical, Torii yakuhin pharma Co.; and Advisory or Leadership Role: Boehringer ingelheim, Bayer pharmaceutical, Otsuka Pharmaceutical Co., Ltd., Nobelpharma Co., Ltd.

I understand that the information above will be published within the journal article, if accepted, and that failure to comply and/or to accurately and completely report the potential financial conflicts of interest could lead to the following: 1) Prior to publication, article rejection, or 2) Post-publication, sanctions ranging from, but not limited to, issuing a correction, reporting the inaccurate information to the authors' institution, banning authors from submitting work to ASN journals for varying lengths of time, and/or retraction of the published work.

Name: Kei Fukami
Manuscript ID: JASN-2026-000059
Manuscript Title: Acute eGFR Changes and Their Mediation of Albuminuria Reduction with Empagliflozin and Finerenone: A CONFIDENCE Trial Analysis
Date of Completion: January 15, 2026
Disclosure Updated Date: January 15, 2026

ASN American Society of Nephrology | JASN Journal of the American Society of Nephrology | KIDNEY360 Accessing Our World From Every Angle | CJASN Clinical Journal of the American Society of Nephrology

# ASN Journal Disclosure Form

As per ASN journal policy, I have disclosed any financial relationships or commitments I have held in the past 36 months as included below. I have listed my Current Employer below to indicate there is a relationship requiring disclosure. If no relationship exists, my Current Employer is not listed.

J. Green reports the following:
Employer: Duke University School of Medicine; Consultancy: AstraZeneca, NovoNordisk, Bayer, Boehringer Ingelheim, Valo, Lilly, Vertex, Corcept, Mineralys, Zealand, Sparrow, Idorsia; Research Funding: Merck, Roche, Lilly, Boehringer Ingelheim, Bluedrop, GentiBio, COUR; Honoraria: Same listing as for consulting: AstraZeneca, NovoNordisk, Bayer, Boehringer Ingelheim, Valo, Lilly, Vertex, Corcept, Mineralys, Zealand, Sparrow, Idorsia; and Advisory or Leadership Role: No payment received; however, I am currently president-elect, medicine and science, of the American Diabetes Association.

I understand that the information above will be published within the journal article, if accepted, and that failure to comply and/or to accurately and completely report the potential financial conflicts of interest could lead to the following: 1) Prior to publication, article rejection, or 2) Post-publication, sanctions ranging from, but not limited to, issuing a correction, reporting the inaccurate information to the authors' institution, banning authors from submitting work to ASN journals for varying lengths of time, and/or retraction of the published work.

Name: Jennifer B. Green
Manuscript ID: JASN-2026-000059
Manuscript Title: Acute eGFR Changes and Their Mediation of Albuminuria Reduction with Empagliflozin and Finerenone: A CONFIDENCE Trial Analysis
Date of Completion: January 15, 2026
Disclosure Updated Date: January 15, 2026

# ASN Journal Disclosure Form

As per ASN journal policy, I have disclosed any financial relationships or commitments I have held in the past 36 months as included below. I have listed my Current Employer below to indicate there is a relationship requiring disclosure. If no relationship exists, my Current Employer is not listed.

H. Heerspink reports the following:
Employer: University Medical Center Groningen; Consultancy: Ongoing consultancy agreements with AstraZeneca, Alexion, Bayer, Boehringer Ingelheim, Biocity Therapeutic, Dimerix, Eli-Lilly, Gilead, Janssen, Novartis, NovoNordisk, Roche, Travere Therapeutics; Research Funding: AstraZeneca, Bayer, Boehringer Ingelheim, NovoNordisk and Janssen research support (grant funding directed to employer); Honoraria: Lecture fees from AstraZeneca and NovoNordisk; and Speakers Bureau: AstraZeneca, Bayer, Novo Nordisk.

I understand that the information above will be published within the journal article, if accepted, and that failure to comply and/or to accurately and completely report the potential financial conflicts of interest could lead to the following: 1) Prior to publication, article rejection, or 2) Post-publication, sanctions ranging from, but not limited to, issuing a correction, reporting the inaccurate information to the authors' institution, banning authors from submitting work to ASN journals for varying lengths of time, and/or retraction of the published work.

Name: Hiddo Jan L. Heerspink
Manuscript ID: JASN-2026-000059
Manuscript Title: Acute eGFR Changes and Their Mediation of Albuminuria Reduction with Empagliflozin and Finerenone: A CONFIDENCE Trial Analysis
Date of Completion: January 27, 2026
Disclosure Updated Date: June 7, 2025

ASN American Society of Nephrology | JASN Journal of the American Society of Nephrology | KIDNEY360 Accessing Our World From Every Angle | CJASN Clinical Journal of the American Society of Nephrology

# ASN Journal Disclosure Form

As per ASN journal policy, I have disclosed any financial relationships or commitments I have held in the past 36 months as included below. I have listed my Current Employer below to indicate there is a relationship requiring disclosure. If no relationship exists, my Current Employer is not listed.

L. Li reports the following:
Employer: Bayer AG; and Ownership Interest: Bayer AG.

I understand that the information above will be published within the journal article, if accepted, and that failure to comply and/or to accurately and completely report the potential financial conflicts of interest could lead to the following: 1) Prior to publication, article rejection, or 2) Post-publication, sanctions ranging from, but not limited to, issuing a correction, reporting the inaccurate information to the authors' institution, banning authors from submitting work to ASN journals for varying lengths of time, and/or retraction of the published work.

Name: Li Li
Manuscript ID: JASN-2026-000059
Manuscript Title: Acute eGFR Changes and Their Mediation of Albuminuria Reduction with Empagliflozin and Finerenone: A CONFIDENCE Trial Analysis
Date of Completion: January 15, 2026
Disclosure Updated Date: July 19, 2025

# ASN Journal Disclosure Form

As per ASN journal policy, I have disclosed any financial relationships or commitments I have held in the past 36 months as included below. I have listed my Current Employer below to indicate there is a relationship requiring disclosure. If no relationship exists, my Current Employer is not listed.

J. Mann reports the following:
Employer: KfH Nierenzentrum; Consultancy: Bayer, Novo Nordisk; Research Funding: Novo Nordisk, Sanofi, Bayer, ICON, Parexel, Cytel, Iqvia, WCG; Honoraria: Bayer, Novo Nordisk, Sanofi, Hexal, ICON, Parexel, Cytel, Iqvia, WCG; Speakers Bureau: Bayer, Hexal, Novo Nordisk; and Other Interests or Relationships: Author for UpToDate.

I understand that the information above will be published within the journal article, if accepted, and that failure to comply and/or to accurately and completely report the potential financial conflicts of interest could lead to the following: 1) Prior to publication, article rejection, or 2) Post-publication, sanctions ranging from, but not limited to, issuing a correction, reporting the inaccurate information to the authors' institution, banning authors from submitting work to ASN journals for varying lengths of time, and/or retraction of the published work.

Name: Johannes F. Mann
Manuscript ID: JASN-2026-000059
Manuscript Title: Acute eGFR Changes and Their Mediation of Albuminuria Reduction with Empagliflozin and Finerenone: A CONFIDENCE Trial Analysis
Date of Completion: January 15, 2026
Disclosure Updated Date: January 9, 2026

ASN American Society of Nephrology JASN Journal of the American Society of Nephrology KIDNEY360 Accessing Our World From Every Angle CJASN Clinical Journal of the American Society of Nephrology

# ASN Journal Disclosure Form

As per ASN journal policy, I have disclosed any financial relationships or commitments I have held in the past 36 months as included below. I have listed my Current Employer below to indicate there is a relationship requiring disclosure. If no relationship exists, my Current Employer is not listed.

J. McGill reports the following:
Employer: Washington University School of Medicine; Consultancy: Bayer, Lilly, Mannkind, NovoNordisk, Pfizer, Vertex; Research Funding: Novo Nordisk, Diamyd, Viking, Cour, Lilly; Patents or Royalties: Washington University; and Other Interests or Relationships: NIH (grant funding, DSMB member); Jaeb Center for Health Resarch (DSMB), Breakthrough T1D (grant funding).

I understand that the information above will be published within the journal article, if accepted, and that failure to comply and/or to accurately and completely report the potential financial conflicts of interest could lead to the following: 1) Prior to publication, article rejection, or 2) Post-publication, sanctions ranging from, but not limited to, issuing a correction, reporting the inaccurate information to the authors' institution, banning authors from submitting work to ASN journals for varying lengths of time, and/or retraction of the published work.

Name: Janet B. McGill
Manuscript ID: JASN-2026-000059
Manuscript Title: Acute eGFR Changes and Their Mediation of Albuminuria Reduction with Empagliflozin and Finerenone: A CONFIDENCE Trial Analysis
Date of Completion: January 27, 2026
Disclosure Updated Date: January 27, 2026

ASN American Society of Nephrology · JASN Journal of the American Society of Nephrology · KIDNEY360 Accessing Our World From Every Angle · CJASN Clinical Journal of the American Society of Nephrology

# ASN Journal Disclosure Form

As per ASN journal policy, I have disclosed any financial relationships or commitments I have held in the past 36 months as included below. I have listed my Current Employer below to indicate there is a relationship requiring disclosure. If no relationship exists, my Current Employer is not listed.

A. Mottl reports the following:
Employer: University of North Carolina at Chapel Hill; Consultancy: Bayer, Chinook, Otsuka, Novartis, Vera; Research Funding: Alexion, Bayer, Boehringer Ingelheim, Chinook, Duke Clinical Research Institute, Novartis, University of Pennsylvania; Honoraria: UpToDate; and Advisory or Leadership Role: Bayer, Chinook, Novartis, Otsuka, Vera.

I understand that the information above will be published within the journal article, if accepted, and that failure to comply and/or to accurately and completely report the potential financial conflicts of interest could lead to the following: 1) Prior to publication, article rejection, or 2) Post-publication, sanctions ranging from, but not limited to, issuing a correction, reporting the inaccurate information to the authors' institution, banning authors from submitting work to ASN journals for varying lengths of time, and/or retraction of the published work.

Name: Amy K. Mottl
Manuscript ID: JASN-2026-000059
Manuscript Title: Acute eGFR Changes and Their Mediation of Albuminuria Reduction with Empagliflozin and Finerenone: A CONFIDENCE Trial Analysis
Date of Completion: January 14, 2026
Disclosure Updated Date: July 18, 2025

# ASN Journal Disclosure Form

As per ASN journal policy, I have disclosed any financial relationships or commitments I have held in the past 36 months as included below. I have listed my Current Employer below to indicate there is a relationship requiring disclosure. If no relationship exists, my Current Employer is not listed.

M. Nangaku reports the following:
Employer: the University of Tokyo Graduate School of Medicine; Consultancy: Kyowa-Kirin, Tanabe-Mitsubishi, Boehringer-Ingelheim; Research Funding: Kyowa-Kirin, Chugai, Boehringer-Ingelheim; and Honoraria: Kyowa-Kirin, Tanabe-Mitsubishi.

I understand that the information above will be published within the journal article, if accepted, and that failure to comply and/or to accurately and completely report the potential financial conflicts of interest could lead to the following: 1) Prior to publication, article rejection, or 2) Post-publication, sanctions ranging from, but not limited to, issuing a correction, reporting the inaccurate information to the authors' institution, banning authors from submitting work to ASN journals for varying lengths of time, and/or retraction of the published work.

Name: Masaomi Nangaku
Manuscript ID: JASN-2026-000059
Manuscript Title: Acute eGFR Changes and Their Mediation of Albuminuria Reduction with Empagliflozin and Finerenone: A CONFIDENCE Trial Analysis
Date of Completion: January 15, 2026
Disclosure Updated Date: May 15, 2025

ASN American Society of Nephrology JASN Journal of the American Society of Nephrology KIDNEY360 Accessing Our World From Every Angle CJASN Clinical Journal of the American Society of Nephrology

# ASN Journal Disclosure Form

As per ASN journal policy, I have disclosed any financial relationships or commitments I have held in the past 36 months as included below. I have listed my Current Employer below to indicate there is a relationship requiring disclosure. If no relationship exists, my Current Employer is not listed.

S. Navaneethan reports the following:
Employer: Baylor College of Medicine and Michael E. DeBakey VA Medical Center; Consultancy: Event adjudication committee- Alnylam, Intercept, ProKidney, Vertex ; Consultant- AstraZeneca, Bayer, Boehringer-Ingelheim, Novartis; Honoraria: Event adjudication committee- Alnylam, Intercept, ProKidney, Vertex ; Consultant- AstraZeneca, Bayer, Boehringer-Ingelheim, Novartis; and Advisory or Leadership Role: AJKD, Current Opinion in Nephrology and Hypertension, American Journal of Nephrology, KDIGO: Guideline writing committee member, ASN- Member, Guidance statement work group, and KDOQI.

I understand that the information above will be published within the journal article, if accepted, and that failure to comply and/or to accurately and completely report the potential financial conflicts of interest could lead to the following: 1) Prior to publication, article rejection, or 2) Post-publication, sanctions ranging from, but not limited to, issuing a correction, reporting the inaccurate information to the authors' institution, banning authors from submitting work to ASN journals for varying lengths of time, and/or retraction of the published work.

Name: Sankar D. Navaneethan
Manuscript ID: JASN-2026-000059
Manuscript Title: Acute eGFR Changes and Their Mediation of Albuminuria Reduction with Empagliflozin and Finerenone: A CONFIDENCE Trial Analysis
Date of Completion: January 14, 2026
Disclosure Updated Date: November 18, 2025

ASN
American Society of Nephrology
JASN
Journal of the American Society of Nephrology
KIDNEY360
Accessing Our World From Every Angle
CJASN
Clinical Journal of the American Society of Nephrology

# ASN Journal Disclosure Form

As per ASN journal policy, I have disclosed any financial relationships or commitments I have held in the past 36 months as included below. I have listed my Current Employer below to indicate there is a relationship requiring disclosure. If no relationship exists, my Current Employer is not listed.

J. Rosenstock reports the following:
Employer: Dallas Diabetes Research Consulting; Consultancy: Amgen, Applied Therapeutics, Biomea Fusion, Boehringer Ingelheim, Eccogene, Eli Lilly, Hanmi, Novo Nordisk, Regeneron, Regor, Roche, Sanofi, Structure Therapeutics, and Terns.; Research Funding: Amgen, Applied Therapeutics, AstraZeneca, Biomea Fusion, Boehringer Ingelheim, Carmot, Corcept, Corxel, Eli Lilly, Hanmi, Merck, Novartis, Novo Nordisk, Pfizer, Regeneron, Roche, Regor, Sanofi, Structure Therapeutics, and Terns.; Honoraria: Novo Nordisk, Sanofi, Eli Lilly; and Speakers Bureau: Novo Nordisk, Eli Lilly.

I understand that the information above will be published within the journal article, if accepted, and that failure to comply and/or to accurately and completely report the potential financial conflicts of interest could lead to the following: 1) Prior to publication, article rejection, or 2) Post-publication, sanctions ranging from, but not limited to, issuing a correction, reporting the inaccurate information to the authors’ institution, banning authors from submitting work to ASN journals for varying lengths of time, and/or retraction of the published work.

Name: Julio Rosenstock
Manuscript ID: JASN-2026-000059
Manuscript Title: Acute eGFR Changes and Their Mediation of Albuminuria Reduction with Empagliflozin and Finerenone: A CONFIDENCE Trial Analysis
Date of Completion: January 15, 2026
Disclosure Updated Date: January 15, 2026

ASN American Society of Nephrology
JASN Journal of the American Society of Nephrology
KIDNEY360 Accessing Our World From Every Angle
CJASN Clinical Journal of the American Society of Nephrology

# ASN Journal Disclosure Form

As per ASN journal policy, I have disclosed any financial relationships or commitments I have held in the past 36 months as included below. I have listed my Current Employer below to indicate there is a relationship requiring disclosure. If no relationship exists, my Current Employer is not listed.

P. Rossing reports the following:
Employer: Steno Diabetes Center Copenhagen; Research Funding: Novo Nordisk , AstraZeneca, Bayer, Lexicon Pharma; Honoraria: Boehringer Ingelheim, AstraZeneca, Abbott, Novo Nordisk, all honoraria to institution; and Advisory or Leadership Role: Astra Zeneca Bayer , Novo Nordisk, Gilead all honoraria to institution.

I understand that the information above will be published within the journal article, if accepted, and that failure to comply and/or to accurately and completely report the potential financial conflicts of interest could lead to the following: 1) Prior to publication, article rejection, or 2) Post-publication, sanctions ranging from, but not limited to, issuing a correction, reporting the inaccurate information to the authors' institution, banning authors from submitting work to ASN journals for varying lengths of time, and/or retraction of the published work.

Name: Peter Rossing
Manuscript ID: manuscript JASN-2026-000059
Manuscript Title: Acute eGFR Changes and Their Mediation of Albuminuria Reduction with Empagliflozin and Finerenone: A CONFIDENCE Trial Analysis,
Date of Completion: January 14, 2026
Disclosure Updated Date: March 19, 2025

ASN American Society of Nephrology | JASN Journal of the American Society of Nephrology | KIDNEY360 Accessing Our World From Every Angle | CJASN Clinical Journal of the American Society of Nephrology

# ASN Journal Disclosure Form

As per ASN journal policy, I have disclosed any financial relationships or commitments I have held in the past 36 months as included below. I have listed my Current Employer below to indicate there is a relationship requiring disclosure. If no relationship exists, my Current Employer is not listed.

C. Scott reports the following:
Employer: Bayer Healthcare Inc

I understand that the information above will be published within the journal article, if accepted, and that failure to comply and/or to accurately and completely report the potential financial conflicts of interest could lead to the following: 1) Prior to publication, article rejection, or 2) Post-publication, sanctions ranging from, but not limited to, issuing a correction, reporting the inaccurate information to the authors' institution, banning authors from submitting work to ASN journals for varying lengths of time, and/or retraction of the published work.

Name: Charlie Scott
Manuscript ID: JASN-2026-000059
Manuscript Title: Acute eGFR Changes and Their Mediation of Albuminuria Reduction with Empagliflozin and Finerenone: A CONFIDENCE Trial Analysis
Date of Completion: January 15, 2026
Disclosure Updated Date: February 19, 2025

# ASN Journal Disclosure Form

As per ASN journal policy, I have disclosed any financial relationships or commitments I have held in the past 36 months as included below. I have listed my Current Employer below to indicate there is a relationship requiring disclosure. If no relationship exists, my Current Employer is not listed.

M. Vaduganathan reports the following:
Employer: Brigham and Women's Hospital, Harvard Medical School; Consultancy: Alnylam Pharmaceuticals, American Regent, Amgen, AstraZeneca, Bayer AG, Baxter Healthcare, BMS, Boehringer Ingelheim, Chiesi, Cytokinetics, Esperion, Fresenius Medical Care, Idorsia Pharmaceuticals, Lexicon Pharmaceuticals, Merck, Milestone Pharmaceuticals, Novartis, Novo Nordisk, Pharmacosmos, Recordati, Relypsa, Roche Diagnostics, Sanofi, and Tricog Health; Research Funding: Amgen, AstraZeneca, Boehringer Ingelheim, Galmed, Novartis, Bayer AG, Occlutech, Pharmacosmos, and Impulse Dynamics.; and Speakers Bureau: AstraZeneca, Bayer AG, Boehringer Ingelheim, Novartis, Roche Diagnostics, Lexicon Pharmaceuticals, Cytokinetics.

I understand that the information above will be published within the journal article, if accepted, and that failure to comply and/or to accurately and completely report the potential financial conflicts of interest could lead to the following: 1) Prior to publication, article rejection, or 2) Post-publication, sanctions ranging from, but not limited to, issuing a correction, reporting the inaccurate information to the authors' institution, banning authors from submitting work to ASN journals for varying lengths of time, and/or retraction of the published work.

Name: Muthiah Vaduganathan
Manuscript ID: JASN-2026-000059R2
Manuscript Title: Acute eGFR Changes and Their Mediation of Albuminuria Reduction with Empagliflozin and Finerenone
Date of Completion: February 27, 2026
Disclosure Updated Date: January 28, 2026

ASN
American Society of Nephrology
JASN
Journal of the American Society of Nephrology
KIDNEY360
Accessing Our World From Every Angle
CJASN
Clinical Journal of the American Society of Nephrology

# ASN Journal Disclosure Form

As per ASN journal policy, I have disclosed any financial relationships or commitments I have held in the past 36 months as included below. I have listed my Current Employer below to indicate there is a relationship requiring disclosure. If no relationship exists, my Current Employer is not listed.

M. Weir reports the following:
Employer: University of Maryland School of Medicine; Consultancy: AstraZeneca, Bayer, Novo Nordisk, Vera, Mineralys, CSL Vifor, Medtronic, Intercept, Daxor , Corcept, Boehringer-Ingelheim; and Honoraria: Same as above for ad hoc advisory board meetings.

I understand that the information above will be published within the journal article, if accepted, and that failure to comply and/or to accurately and completely report the potential financial conflicts of interest could lead to the following: 1) Prior to publication, article rejection, or 2) Post-publication, sanctions ranging from, but not limited to, issuing a correction, reporting the inaccurate information to the authors' institution, banning authors from submitting work to ASN journals for varying lengths of time, and/or retraction of the published work.

Name: Matthew R. Weir
Manuscript ID: JASN-2026-000059
Manuscript Title: Acute eGFR changes and their mediation of albuminuria reduction with empagliflozin and finerenone: A CONFIDENCE trial analysis
Date of Completion: January 14, 2026
Disclosure Updated Date: January 14, 2026
