# Supplementary material for: Acute eGFR Changes and Their Mediation of Albuminuria Reduction with Empagliflozin and Finerenone
Source: J Am Soc Nephrol. 2026 Mar 29;37(9):1969–78. doi: 10.1681/ASN.0000001071 (PMC13567893; doi:10.1681/ASN.0000001071)
Supplement: Supplementary file 2 [file jasn-37-1969-s002.pdf]

Supplemental Material

Supplemental Figure 1. eGFR decline as a mediator of UACR lowering.

Supplemental Figure 2. Participants with eGFR decline ≥30% from baseline.

Supplemental Table 1: Change in estimated glomerular filtration rate from baseline within each treatment group.

Supplemental Table 2: Change in estimated glomerular filtration rate from baseline within each treatment group by UACR.

Supplemental Table 3: Change in eGFR from baseline within each treatment group by eGFR.

Supplemental Table 4: Change in eGFR from baseline within each treatment group by systolic blood pressure (BP).

Supplemental Table 5: Change in eGFR from baseline within each treatment group by diuretic use at baseline.

Supplemental Figure 1. eGFR decline as a mediator of UACR lowering.

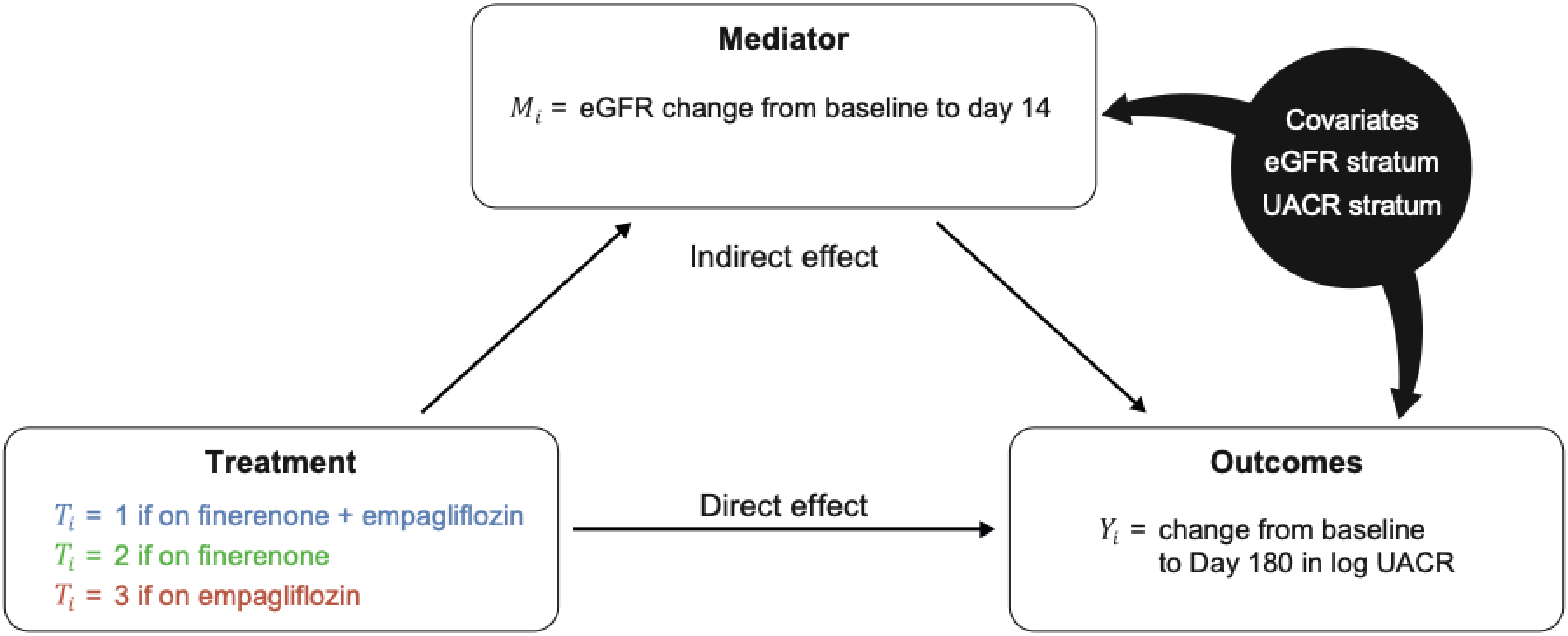

eGFR, estimated glomerular filtration rate, UACR, urinary albumin-to-creatinine ratio.

Supplemental Figure 2. Participants with eGFR decline ≥30% from baseline.

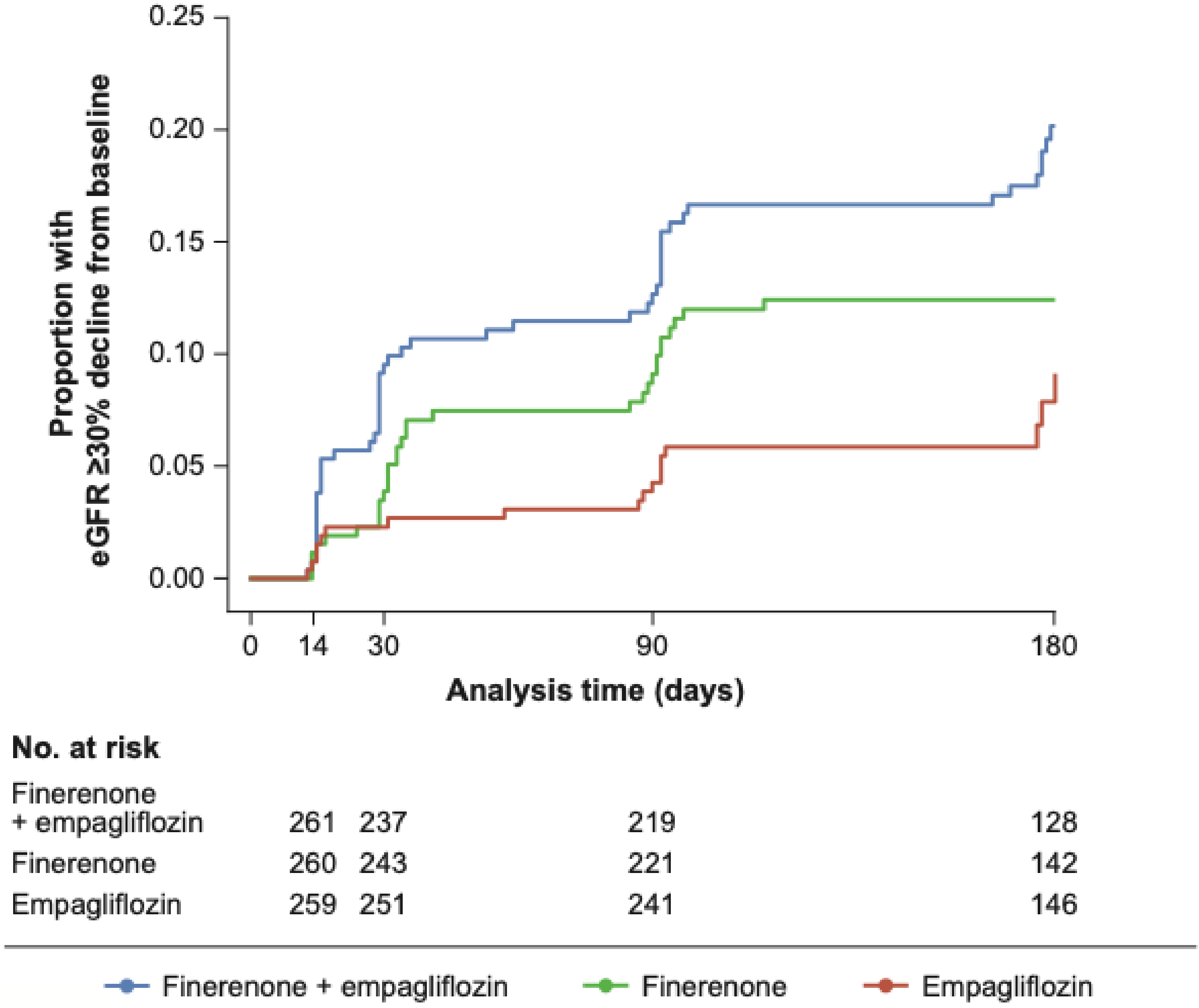

eGFR, estimated glomerular filtration rate.

Supplemental Table 1: Change in estimated glomerular filtration rate from baseline within each treatment group

Treatment	Day	eGFR change from baseline (95% CI)
Combination	14	-6.6 (-7.7 to -5.6)
Combination	30	-6.2 (-7.3 to -5.2)
Combination	90	-6.0 (-7.1 to -4.9)
Combination	180	-5.6 (-6.7 to -4.4)
Combination	210	-1.7 (-2.9 to -0.5)
Finerenone	14	-2.1 (-3.2 to -1.1)
Finerenone	30	-2.7 (-3.8 to -1.7)
Finerenone	90	-4.4 (-5.5 to -3.3)
Finerenone	180	-4.2 (-5.4 to -3.0)
Finerenone	210	-3.1 (-4.4 to -1.9)
Empagliflozin	14	-4.8 (-5.9 to -3.8)
Empagliflozin	30	-4.6 (-5.7 to -3.5)
Empagliflozin	90	-3.9 (-5.0 to -2.8)
Empagliflozin	180	-4.1 (-5.3 to -2.9)
Empagliflozin	210	-1.7 (-2.9 to -0.5)

eGFR = estimated glomerular filtration rate, (mL/min/1.73m^2)

Supplemental Table 2: Change in estimated glomerular filtration rate from baseline within each treatment group by UACR

Treatment	Day	UACR	eGFR change from baseline (95% CI)
Combination	14	100	-6.3 (-7.8 to -4.8)
Combination	14	300	-6.6 (-7.7 to -5.4)
Combination	14	1000	-6.9 (-8.0 to -5.7)
Combination	14	3000	-7.1 (-8.6 to -5.6)
Combination	30	100	-5.3 (-6.8 to -3.8)
Combination	30	300	-5.9 (-7.1 to -4.8)
Combination	30	1000	-6.6 (-7.7 to -5.5)
Combination	30	3000	-7.2 (-8.7 to -5.7)
Combination	90	100	-4.1 (-5.6 to -2.5)
Combination	90	300	-5.3 (-6.5 to -4.1)
Combination	90	1000	-6.6 (-7.8 to -5.5)
Combination	90	3000	-7.9 (-9.4 to -6.3)
Combination	180	100	-3.4 (-5.1 to -1.8)
Combination	180	300	-4.8 (-6.0 to -3.6)
Combination	180	1000	-6.3 (-7.6 to -5.1)
Combination	180	3000	-7.7 (-9.4 to -6.1)
Combination	210	100	1.5 (-0.2 to 3.3)
Combination	210	300	-0.5 (-1.8 to 0.8)
Combination	210	1000	-2.8 (-4.0 to -1.5)
Combination	210	3000	-4.8 (-6.6 to -3.1)
Finerenone	14	100	-1.7 (-3.3 to -0.2)
Finerenone	14	300	-2.0 (-3.2 to -0.9)
Finerenone	14	1000	-2.3 (-3.4 to -1.2)
Finerenone	14	3000	-2.6 (-4.0 to -1.1)
Finerenone	30	100	-1.8 (-3.4 to -0.2)
Finerenone	30	300	-2.4 (-3.6 to -1.3)
Finerenone	30	1000	-3.1 (-4.2 to -2.0)
Finerenone	30	3000	-3.7 (-5.2 to -2.2)
Finerenone	90	100	-2.5 (-4.1 to -0.9)
Finerenone	90	300	-3.8 (-5.0 to -2.6)
Finerenone	90	1000	-5.1 (-6.3 to -3.9)
Finerenone	90	3000	-6.3 (-7.9 to -4.8)
Finerenone	180	100	-2.0 (-3.7 to -0.3)
Finerenone	180	300	-3.4 (-4.7 to -2.2)
Finerenone	180	1000	-5.0 (-6.2 to -3.7)
Finerenone	180	3000	-6.3 (-8.0 to -4.7)
Finerenone	210	100	0.1 (-1.7 to 1.9)
Finerenone	210	300	-2.0 (-3.3 to -0.6)
Finerenone	210	1000	-4.2 (-5.5 to -2.9)
Finerenone	210	3000	-6.3 (-8.0 to -4.6)
Empagliflozin	14	100	-4.3 (-5.8 to -2.7)
Empagliflozin	14	300	-4.5 (-5.7 to -3.4)
Empagliflozin	14	1000	-4.8 (-5.9 to -3.7)

Empagliflozin	14	3000	-5.1 (-6.6 to -3.6)
Empagliflozin	30	100	-3.5 (-5.1 to -2.0)
Empagliflozin	30	300	-4.1 (-5.3 to -3.0)
Empagliflozin	30	1000	-4.8 (-5.9 to -3.7)
Empagliflozin	30	3000	-5.4 (-6.9 to -3.9)
Empagliflozin	90	100	-1.8 (-3.4 to -0.2)
Empagliflozin	90	300	-3.1 (-4.3 to -1.9)
Empagliflozin	90	1000	-4.4 (-5.6 to -3.2)
Empagliflozin	90	3000	-5.6 (-7.2 to -4.1)
Empagliflozin	180	100	-1.8 (-3.4 to -0.1)
Empagliflozin	180	300	-3.2 (-4.4 to -1.9)
Empagliflozin	180	1000	-4.7 (-5.9 to -3.5)
Empagliflozin	180	3000	-6.1 (-7.7 to -4.4)
Empagliflozin	210	100	1.6 (-0.1 to 3.4)
Empagliflozin	210	300	-0.4 (-1.7 to 0.9)
Empagliflozin	210	1000	-2.7 (-4.0 to -1.4)
Empagliflozin	210	3000	-4.7 (-6.4 to -3.0)

UACR: urine albumin to urine creatinine ration (mg/g)

eGFR: estimated glomerular filtration rate, (mL/min/1.73m^2)

Supplemental Table 3: Change in eGFR from baseline within each treatment group by eGFR

Treatment	Day	eGFR	eGFR change from baseline (95% CI)
Combination	14	30	-1.4 (-2.8 to 0.0)
Combination	14	45	-4.7 (-5.8 to -3.6)
Combination	14	60	-8.0 (-9.1 to -6.9)
Combination	14	75	-11.3 (-12.6 to -10.0)
Combination	30	30	-0.3 (-1.7 to 1.1)
Combination	30	45	-4.0 (-5.1 to -2.9)
Combination	30	60	-7.7 (-8.8 to -6.6)
Combination	30	75	-11.4 (-12.7 to -10.1)
Combination	90	30	0.0 (-1.4 to 1.4)
Combination	90	45	-3.7 (-4.9 to -2.6)
Combination	90	60	-7.4 (-8.5 to -6.3)
Combination	90	75	-11.1 (-12.4 to -9.8)
Combination	180	30	0.2 (-1.3 to 1.7)
Combination	180	45	-3.4 (-4.6 to -2.2)
Combination	180	60	-7.0 (-8.1 to -5.8)
Combination	180	75	-10.6 (-12.0 to -9.1)
Combination	210	30	2.7 (1.2 to 4.3)
Combination	210	45	0.0 (-1.2 to 1.3)
Combination	210	60	-2.7 (-3.9 to -1.5)
Combination	210	75	-5.4 (-6.9 to -3.9)
Finerenone	14	30	3.2 (1.8 to 4.6)
Finerenone	14	45	-0.1 (-1.2 to 1.0)
Finerenone	14	60	-3.4 (-4.5 to -2.3)
Finerenone	14	75	-6.7 (-8.0 to -5.4)
Finerenone	30	30	3.2 (1.8 to 4.6)
Finerenone	30	45	-0.5 (-1.6 to 0.6)
Finerenone	30	60	-4.2 (-5.3 to -3.1)
Finerenone	30	75	-7.9 (-9.2 to -6.6)
Finerenone	90	30	1.5 (0.1 to 2.9)
Finerenone	90	45	-2.2 (-3.4 to -1.0)
Finerenone	90	60	-5.9 (-7.0 to -4.8)
Finerenone	90	75	-9.6 (-10.9 to -8.2)
Finerenone	180	30	1.5 (0.0 to 3.1)
Finerenone	180	45	-2.0 (-3.3 to -0.8)
Finerenone	180	60	-5.6 (-6.8 to -4.4)
Finerenone	180	75	-9.2 (-10.6 to -7.8)
Finerenone	210	30	1.2 (-0.4 to 2.8)
Finerenone	210	45	-1.5 (-2.8 to -0.2)
Finerenone	210	60	-4.2 (-5.5 to -3.0)
Finerenone	210	75	-7.0 (-8.5 to -5.5)
Empagliflozin	14	30	0.6 (-0.8 to 2.0)
Empagliflozin	14	45	-2.7 (-3.8 to -1.6)
Empagliflozin	14	60	-6.0 (-7.1 to -4.9)

Empagliflozin	14	75	-9.3 (-10.6 to -8.0)
Empagliflozin	30	30	1.5 (0.1 to 2.8)
Empagliflozin	30	45	-2.2 (-3.4 to -1.1)
Empagliflozin	30	60	-5.9 (-7.0 to -4.9)
Empagliflozin	30	75	-9.7 (-11.0 to -8.3)
Empagliflozin	90	30	2.1 (0.7 to 3.6)
Empagliflozin	90	45	-1.6 (-2.7 to -0.4)
Empagliflozin	90	60	-5.3 (-6.4 to -4.1)
Empagliflozin	90	75	-9.0 (-10.3 to -7.6)
Empagliflozin	180	30	1.8 (0.3 to 3.3)
Empagliflozin	180	45	-1.8 (-3.0 to -0.6)
Empagliflozin	180	60	-5.4 (-6.6 to -4.2)
Empagliflozin	180	75	-9.0 (-10.4 to -7.6)
Empagliflozin	210	30	2.7 (1.1 to 4.3)
Empagliflozin	210	45	0.0 (-1.3 to 1.3)
Empagliflozin	210	60	-2.7 (-4.0 to -1.5)
Empagliflozin	210	75	-5.4 (-6.9 to -3.9)

eGFR: estimated glomerular filtration rate, (mL/min/1.73m^2)

Supplemental Table 4: Change in eGFR from baseline within each treatment group by systolic blood pressure (BP)

Treatment	Day	systolic BP (mmHg)	eGFR change from baseline (95% CI)
Combination	14	120	-6.0 (-7.3 to -4.7)
Combination	14	130	-6.5 (-7.6 to -5.4)
Combination	14	140	-6.9 (-8.0 to -5.9)
Combination	14	150	-7.4 (-8.7 to -6.1)
Combination	30	120	-5.2 (-6.5 to -3.9)
Combination	30	130	-5.9 (-7.0 to -4.8)
Combination	30	140	-6.6 (-7.7 to -5.5)
Combination	30	150	-7.3 (-8.6 to -6.1)
Combination	90	120	-5.3 (-6.6 to -4.0)
Combination	90	130	-5.7 (-6.9 to -4.6)
Combination	90	140	-6.2 (-7.3 to -5.1)
Combination	90	150	-6.6 (-8.0 to -5.3)
Combination	180	120	-4.5 (-5.9 to -3.1)
Combination	180	130	-5.2 (-6.4 to -4.0)
Combination	180	140	-5.9 (-7.1 to -4.8)
Combination	180	150	-6.6 (-8.0 to -5.3)
Combination	210	120	-0.8 (-2.2 to 0.7)
Combination	210	130	-1.3 (-2.6 to -0.1)
Combination	210	140	-1.9 (-3.1 to -0.7)
Combination	210	150	-2.5 (-3.9 to -1.0)
Finerenone	14	120	-1.5 (-2.7 to -0.2)
Finerenone	14	130	-1.9 (-3.0 to -0.9)
Finerenone	14	140	-2.4 (-3.5 to -1.3)
Finerenone	14	150	-2.9 (-4.2 to -1.6)
Finerenone	30	120	-1.7 (-3.0 to -0.5)
Finerenone	30	130	-2.5 (-3.5 to -1.4)
Finerenone	30	140	-3.2 (-4.3 to -2.1)
Finerenone	30	150	-3.9 (-5.2 to -2.6)
Finerenone	90	120	-3.8 (-5.2 to -2.5)
Finerenone	90	130	-4.3 (-5.4 to -3.2)
Finerenone	90	140	-4.7 (-5.9 to -3.6)
Finerenone	90	150	-5.2 (-6.5 to -3.9)
Finerenone	180	120	-3.2 (-4.6 to -1.8)
Finerenone	180	130	-3.9 (-5.1 to -2.7)
Finerenone	180	140	-4.6 (-5.8 to -3.4)
Finerenone	180	150	-5.4 (-6.8 to -4.0)
Finerenone	210	120	-2.4 (-3.9 to -0.9)
Finerenone	210	130	-3.0 (-4.2 to -1.7)
Finerenone	210	140	-3.5 (-4.8 to -2.2)
Finerenone	210	150	-4.1 (-5.6 to -2.6)
Empagliflozin	14	120	-4.0 (-5.3 to -2.7)
Empagliflozin	14	130	-4.5 (-5.5 to -3.4)
Empagliflozin	14	140	-4.9 (-6.0 to -3.8)

Empagliflozin	14	150	-5.4 (-6.7 to -4.1)
Empagliflozin	30	120	-3.4 (-4.7 to -2.1)
Empagliflozin	30	130	-4.1 (-5.2 to -3.0)
Empagliflozin	30	140	-4.8 (-5.9 to -3.7)
Empagliflozin	30	150	-5.6 (-6.8 to -4.3)
Empagliflozin	90	120	-3.1 (-4.5 to -1.8)
Empagliflozin	90	130	-3.6 (-4.7 to -2.4)
Empagliflozin	90	140	-4.0 (-5.1 to -2.9)
Empagliflozin	90	150	-4.5 (-5.8 to -3.1)
Empagliflozin	180	120	-2.9 (-4.3 to -1.5)
Empagliflozin	180	130	-3.6 (-4.8 to -2.4)
Empagliflozin	180	140	-4.3 (-5.5 to -3.1)
Empagliflozin	180	150	-5.0 (-6.4 to -3.7)
Empagliflozin	210	120	-0.8 (-2.3 to 0.7)
Empagliflozin	210	130	-1.4 (-2.6 to -0.1)
Empagliflozin	210	140	-1.9 (-3.2 to -0.7)
Empagliflozin	210	150	-2.5 (-3.9 to -1.0)

eGFR: estimated glomerular filtration rate, (mL/min/1.73m^2)

Supplemental Table 5: Change in eGFR from baseline within each treatment group by diuretic use at baseline

Treatment	Day	Diuretic use	eGFR change from baseline (95% CI)
Combination	14	No	-5.8 (-7.0 to -4.6)
Combination	14	Yes	-8.0 (-9.3 to -6.7)
Combination	30	No	-5.6 (-6.8 to -4.4)
Combination	30	Yes	-7.2 (-8.5 to -5.9)
Combination	90	No	-5.4 (-6.6 to -4.1)
Combination	90	Yes	-6.9 (-8.2 to -5.5)
Combination	180	No	-4.6 (-5.9 to -3.4)
Combination	180	Yes	-7.0 (-8.4 to -5.5)
Combination	210	No	-1.2 (-2.5 to 0.2)
Combination	210	Yes	-2.3 (-3.8 to -0.8)
Finerenone	14	No	-1.4 (-2.5 to -0.2)
Finerenone	14	Yes	-3.6 (-4.9 to -2.2)
Finerenone	30	No	-2.2 (-3.4 to -1.0)
Finerenone	30	Yes	-3.8 (-5.2 to -2.5)
Finerenone	90	No	-4.0 (-5.2 to -2.7)
Finerenone	90	Yes	-5.5 (-6.9 to -4.1)
Finerenone	180	No	-3.4 (-4.7 to -2.2)
Finerenone	180	Yes	-5.7 (-7.2 to -4.3)
Finerenone	210	No	-2.8 (-4.2 to -1.5)
Finerenone	210	Yes	-3.9 (-5.5 to -2.4)
Empagliflozin	14	No	-4.0 (-5.1 to -2.9)
Empagliflozin	14	Yes	-6.2 (-7.6 to -4.8)
Empagliflozin	30	No	-4.0 (-5.1 to -2.8)
Empagliflozin	30	Yes	-5.6 (-7.0 to -4.2)
Empagliflozin	90	No	-3.3 (-4.5 to -2.1)
Empagliflozin	90	Yes	-4.8 (-6.3 to -3.4)
Empagliflozin	180	No	-3.3 (-4.5 to -2.0)
Empagliflozin	180	Yes	-5.6 (-7.1 to -4.1)
Empagliflozin	210	No	-1.3 (-2.6 to 0.0)
Empagliflozin	210	Yes	-2.4 (-4.0 to -0.8)

eGFR: estimated glomerular filtration rate, (mL/min/1.73m^2)
